# Activation of PPARα by Fenofibrate Attenuates the Effect of Local Heart High Dose Irradiation on the Mouse Cardiac Proteome

**DOI:** 10.3390/biomedicines9121845

**Published:** 2021-12-06

**Authors:** Omid Azimzadeh, Vikram Subramanian, Wolfgang Sievert, Juliane Merl-Pham, Kateryna Oleksenko, Michael Rosemann, Gabriele Multhoff, Michael J. Atkinson, Soile Tapio

**Affiliations:** 1 Section Radiation Biology, Federal Office for Radiation Protection, 85764 Neuherberg, Germany; 2 Institute of Radiation Biology, Helmholtz Zentrum München—German Research Center for Environmental Health GmbH, 85764 Neuherberg, Germany; vikram-subramanian@uiowa.edu (V.S.); kateryna.oleksenko97@gmail.com (K.O.); rosemann@helmholtz-muenchen.de (M.R.); michaeljatkinson@icloud.com (M.J.A.); soile.tapio@helmholtz-muenchen.de (S.T.); 3 Abboud Cardiovascular Research Center, Division of Cardiovascular Medicine, Department of Internal Medicine, Carver College of Medicine, University of Iowa, Iowa, IA 52242, USA; 4 Department of Radiation Oncology, Campus Klinikum rechts der Isar, Technical University of Munich (TUM), 81675 Munich, Germany; wolfgang.sievert@tum.de (W.S.); gabriele.multhoff@tum.de (G.M.); 5 Central Institute for Translational Cancer Research-TranslaTUM, Klinikum rechts der Isar, Technical University of Munich (TUM), 81675 Munich, Germany; 6 Research Unit Protein Science, Helmholtz Zentrum München—German Research Centre for Environmental Health GmbH, 80939 Munich, Germany; juliane.merl@helmholtz-muenchen.de; 7 Chair of Radiation Biology, School of Medicine, Technical University of Munich (TUM), 81675 Munich, Germany; 8 Institute of Biological and Medical Imaging, Helmholtz Zentrum München—German Research Center for Environmental Health GmbH, 85764 Neuherberg, Germany

**Keywords:** ionizing radiation, proteomics, label-free quantification, PPARα, fenofibrate, agonist, TGF-β, cardiac metabolism, NO, cardiovascular disease

## Abstract

Radiation-induced cardiovascular disease is associated with metabolic remodeling in the heart, mainly due to the inactivation of the transcription factor peroxisome proliferator-activated receptor alpha (PPARα), thereby inhibiting lipid metabolic enzymes. The objective of the present study was to investigate the potential protective effect of fenofibrate, a known agonist of PPARα on radiation-induced cardiac toxicity. To this end, we compared, for the first time, the cardiac proteome of fenofibrate- and placebo-treated mice 20 weeks after local heart irradiation (16 Gy) using label-free proteomics. The observations were further validated using immunoblotting, enzyme activity assays, and ELISA. The analysis showed that fenofibrate restored signalling pathways that were negatively affected by irradiation, including lipid metabolism, mitochondrial respiratory chain, redox response, tissue homeostasis, endothelial NO signalling and the inflammatory status. The results presented here indicate that PPARα activation by fenofibrate attenuates the cardiac proteome alterations induced by irradiation. These findings suggest a potential benefit of fenofibrate administration in the prevention of cardiovascular diseases, following radiation exposure.

## 1. Introduction

Exposure to high-dose ionizing radiation is strongly associated with the development of cardiovascular disease (CVD) as observed in patients after thoracic radiotherapy treatment [1,2,3,4]. Patients treated with radiotherapy for left-sided breast cancer were found to have a significant increase in mortality from CVD compared to those treated for right-sided cancer [1,5,6,7]. Radiation-induced cardiac toxicity is accompanied by a persistent perturbation of lipid metabolism [8,9,10] through the deactivation of its key regulator, peroxisome proliferator-activated receptor alpha (PPARα) [11,12,13].

PPARα is a ligand-activated transcription factor that is crucial for energy production and is therefore strongly expressed in tissues with high metabolic activity. In cardiac tissue, it plays an essential regulatory role in fatty acid oxidation, lipoprotein assembly, and lipid transport [14,15,16] and also in anti-inflammatory responses [17,18]. The impairment of the lipid metabolism has been described as a consequence of the reduced transcriptional activity of PPARα [15]. PPARα-null mice show a characteristically low expression of proteins involved in fatty acid metabolism and transport [16,18].

The transcriptional activity of PPARα depends to a great extent on the availability of exogenous (fibrates) and endogenous (fatty acids and their derivatives) ligands [19]. Upon binding to the ligand, PPARα changes its conformation and heterodimerizes with its coactivator, PPAR gamma coactivator-1 (PGC-1) and retinoid X receptor (RXR), to form the transcriptional complex. This complex recognizes PPAR response elements (PPRE) in promoters of the target genes, resulting in their transcription [20,21,22]. Synthetic PPARα agonists are therefore attractive candidates for designing novel therapeutic measures for cardiac disease [21,23]. As there is accruing evidence showing that PPARα is a particular target for ionizing radiation in the heart [8,24], it is reasonable to assume that its activation, using ligands such as fenofibrate, could alleviate radiation-associated heart damage.

Consequently, the present study aimed to investigate whether the fenofibrate-activation of PPARα is able to protect the heart against the late effects of high-dose irradiation. To address this question, we examined the effects of radiation exposure on the cardiac proteome of fenofibrate- and placebo-treated mice. Indeed, the findings presented in this study suggest that activation of the PPARα pathway using fenofibrate has a positive impact on the outcome of radiation-induced cardiac damage.

## 2. Experimental Section

### 2.1. Methods

#### 2.1.1. Irradiation and Fenofibrate Treatment

Male C57BL/6J mice 8 weeks of age were purchased from Charles River Germany (Sulzbach, Germany) and kept at the Helmholtz Zentrum München SPF facility. Mice in groups of five were housed in individually ventilated racks and provided food and water ad libitum. Fenofibrate was administered orally for 4 weeks (2 weeks before and 2 weeks after irradiation) using a commercially prepared fenofibrate diet, ensuring a daily uptake of 100 mg/kg body weight. The dose of fenofibrate that is used for the current experiment set up has been previously shown to have no toxic effect at the selected concentration [25,26]. The control animals were fed with a placebo. Local heart irradiation was carried out as previously described [12]. Briefly, mice were irradiated with a single X-ray dose of 16 Gy locally to the heart (200 kV, 10 mA) (Gulmay, Camberley, UK). The age-matched control mice were sham irradiated. Mice were not anaesthetized during irradiation but were held in a prone position in restraining jigs with thorax fixed using adjustable hinges. The position and field size (9 × 13 mm^2^) of the heart were determined by pilot studies soft X-rays; the rest of the body was shielded with a 2 mm thick lead plate. The radiation field by necessity included approximately 40% of the lung volume [27]. The animals were sacrificed 20 weeks after irradiation and hearts and serum were collected and stored at −20 °C for further experiments. All animal experiments were approved and licensed under Bavarian federal law (Certificate No. AZ 55.2-1-54-2532-114-2014). In total, 20 mice were used in this study, with 5 mice in each group. Male animals were used so the results of this study could be compared with results from our earlier studies [11,12,13,28].

#### 2.1.2. Proteome Profiling

The heart samples obtained from at least 5 mice per group were grounded to a fine powder with a cold mortar and pestle before being suspended in a lysis buffer (SERVA) [11]. Protein concentration was determined by the Bradford assay following the manufacturer’s instructions (ThermoFisher, Wilmington, MA, USA).

Protein lysates (10 µg) were digested using a modified filter-aided sample preparation (FASP) protocol [29] After reduction and alkylation using DTT and IAA, the proteins were centrifuged on a 30 kDa cutoff filter device (Sartorius), washed thrice with a UA buffer (8 M urea in 0.1 M Tris/HCl pH 8.5) and twice with 50 mM ammonium bicarbonate. The proteins were digested for 2 h at room temperature using 0.5 µg Lys-C (Wako Chemicals, Neuss, Germany) and for 16 h at 37 °C using 1 µg trypsin (Promega, Mannheim, Germany). After centrifugation (10 min at 14,000× *g*), the eluted peptides were acidified with 0.5% TFA and stored at −20 °C.

An LC-MS/MS analysis was performed on a Q-Exactive HF mass spectrometer (Thermo Scientific) online, coupled to an Ultimate 3000 nano-RSLC (Dionex). Tryptic peptides were automatically loaded on a C18 trap column (300 µm inner diameter (ID) × 5 mm, Acclaim PepMap100 C18, 5 µm, 100 Å, LC Packings) at a 30 µL/min flow rate prior to C18 reversed-phase chromatography on the analytical column (nanoEase MZ HSS T3 Column, 100 Å, 1.8 µm, 75 µm × 250 mm, Waters) at a 250 nL/min flow rate with a 95 min non-linear acetonitrile gradient from 3 to 40% in 0.1% formic acid. Profile precursor spectra from 300 to 1500 *m*/*z* were recorded at a 60,000 resolution with automatic gain control (AGC) target of 3e6 and a maximum injection time of 50 ms. The TOP10 fragment spectra of charges 2 to 7 were recorded at 15,000 resolution with an AGC target of 1e5, a maximum injection time of 50 ms, an isolation window of 1.6 *m*/*z*, a normalized collision energy of 27 and a dynamic exclusion of 30 s. The generated raw files were analyzed using Progenesis QI for proteomics (version 4.0, Nonlinear Dynamics, part of Waters) for label-free quantification as described previously [30,31]. Features of charges 2–7 were used and all MSMS spectra were exported as mgf files. A peptide search was performed using the Mascot search engine (version 2.6.2) against the Swissprot mouse protein database (16,872 sequences, 9,512,882 residues). Search settings were as follows: 10 ppm precursor tolerance, 0.02 Da fragment tolerance, one missed cleavage allowed. Carbamidomethyl on cysteine was set as fixed modification, and the deamidation of glutamine and asparagine were used for variable modification, as well as the oxidation of methionine. Applying the percolator algorithm resulted in a peptide false discovery rate (FDR) of 0.36%. Search results were reimported in the Progenesis QI software. Proteins were quantified by calculating the abundances of all unique peptides per protein. The resultant normalized-protein abundances were used for the calculation of fold-changes and statistical values were exported from the Progenesis QI software.

For final quantifications, proteins identified with more than one unique peptide and ratios of greater than 1.30-fold or less than 0.77-fold (*p*-value ≤ 0.05) were defined as significantly differentially expressed.

#### 2.1.3. Interaction and Signalling Network Analysis

The significantly up- and down-regulated proteins (ratios greater than 1.30-fold or less than 0.77-fold and *p*-value ≤ 0.05) were subjected to protein interaction and function analysis. The signalling networks were analyzed using an Ingenuity^®^ Pathway Analysis. Available online: https://www.qiagenbioinformatics.com/products/ingenuity-pathway-analysis, (accessed on 14 October 2021) [32] and software tool STRING version 11. Available online:(https://string-db.org (accessed on 14 October 2021) [33].

The pathway analysis and prediction of upstream regulators were performed by an exposure of significantly deregulated proteins in the present study to the ingenuity database of curated disease omics data. The protein functional clusters were generated by their exposure of significantly deregulated proteins to the biological database of STRING containing experimental data and public text collections. The proteins that contribute jointly to a specific biological function or cellular compartments were clustered together.

#### 2.1.4. Immunoblot Analysis

The immunoblot analysis was performed as described previously [13] using anti-PPARα (bs-3614R; ThermoFisher, Wilmington, MA, USA), anti-phospho-PPARα (Ser12) (ab3484; Abcam; Boston, MA, USA), anti-SMA (ab32575; Abcam; Boston, MA, USA), anti-PGC1 (ab54481; Abcam; Boston, MA, USA), anti-SIRT3 (#5490; Cell Signaling Technology; Danvers, MA, USA), anti-NRF2 (ab89443; Abcam; Boston, MA, USA), anti-KEAP1 (ab166721; Abcam; Boston, MA, USA), anti-collagen I (ab270993; Abcam; Boston, MA, USA), anti-biglycan (sc-100857; Santa Cruz Biotechnologies; Dallas, TX, USA), anti-fibronectin (sc-8422; Santa Cruz Biotechnologies; Dallas, TX, USA), eNOS (#9572, Cell Signaling Technology, Danvers, MA, USA) and phospho-eNOS (Ser1177) (#9571, Cell Signaling Technology; Danvers, MA, USA). After washing three times, the blots were incubated with the appropriate horseradish peroxidase-conjugated or alkaline phosphatase-conjugated anti-mouse, anti-rabbit or anti-goat secondary antibody (Santa Cruz Biotechnology; Dallas, TX, USA) for 2 h at room temperature and then developed using the SuperSignal West Pico PLUS ECL system (ThermoFisher, Wilmington, MA, USA) or 1-stepTM NBT/BCIP substrate solution (ThermoFisher, Wilmington, MA, USA) following standard procedures. Reversible Ponceau S staining was used as a loading control as the usual loading controls GAPDH, ATP5B, or tubulin showed altered levels of expression in at least one condition based on the proteomics data. The corresponding Ponceau-s stained membrane for each antibody detection was provided in Supplementary Figures S1–S5. Chemiluminescence images of membranes were acquired using ChemiDoc imaging system (BioRad, Bayern, Germany). Digitized images of immunoblot bands from four biological replicates were quantified using ImageJ 1.50f3 software. Available online: http://rsbweb.nih.gov/ij/ (accessed on 14 October 2021).

#### 2.1.5. Serum Free Fatty Acid Assay

The levels of circulating free fatty acids (FFA) (ab65341, Abcam; Boston, MA, USA) were measured according to the manufacturer’s instructions. Briefly, 100 µg of serum proteins (in 50 µL) were incubated with Acyl-CoA Synthetase (ACS) reagent for 30 min at 37 °C. Each reaction was mixed with 50 μL reaction mix containing an assay buffer, fatty acid probe and enhancer and was incubated at 37 °C for 30 min in dark. The output was read as 570 nm. In the assay, fatty acids were converted to coenzyme A, oxidized, and formed a measurable colour/fluorescence.

#### 2.1.6. Complex I Activity Assay

Complex I activity was measured using the assay kit (ab109721, Abcam; Boston, MA, USA) according to the manufacturer’s recommendations. Briefly, the replicates of 100 µg protein of tissue lysate (final concentration of 5.5 mg/mL) were extracted and incubated for 3 h at room temperature in the microplate wells precoated with capture antibodies specific for Complex I. The wells were washed and incubated using the assay solution before measurement at OD 450 nm. The Complex I activity was determined based on the oxidation of NADH to NAD+ and reduction of a dye which led to increased absorbance.

#### 2.1.7. ATP Assay

The ATP levels were measured in the frozen heart samples using a commercially available kit (ab83355, Abcam; Boston, MA, USA) as recommended by the manufacturer. Briefly, the replicates of 100 µg protein lysate diluted in a final volume of 50 µL were mixed with an ATP reaction mix and incubated for 30 min at room temperature. The microplate was analyzed using a reader at OD 570 nm. The assay is based on the phosphorylation of glycerol to generate a product that can be quantified.

#### 2.1.8. Lipid Peroxidation Assay

Stress-induced protein modification was measured using the lipid peroxidation colorimetric assays (ab118970; Abcam; Boston, MA, USA) according to the manufacturer’s instructions. The assay detects malondialdehyde (MDA) as a marker of lipid peroxidation. Briefly, the protein was extracted from heart tissue using an MDA lysis buffer. Replicates of 100 µg (in a final volume of 200 µL) was incubated with thiobarbituric acid (TBA) 95 °C for 1 h. The reaction was cooled to room temperature in an ice bath for 10 min. The absorbance was measured on a microplate reader at OD 532 nm.

#### 2.1.9. SOD Activity Assay

Superoxide dismutase activity was measured in tissue using a colorimetric assay (ab65354, Abcam; Boston, MA, USA) as recommended by the manufacturer. Briefly, the replicates of 100 µg protein lysate (in 20 µL each) were incubated with 200 µL WST-1 (4-[3-(4-iodophenyl)-2-(4-nitrophenyl)-2H-5-tetrazolio]-1,3-benzene disulfonate sodium salt) solution at 37 °C for 20 min and their output was measured at OD 450 nm.

#### 2.1.10. SMAD and Phospho-SMAD Assay

The expression levels of SMAD 2/3 and phospho-SMAD2 (Ser465/467) / phosphor-SMAD3 (Ser423/425) were measured using PathScan^®^ Total Smad2/3 and phosphor Smad Sandwich ELISA Kit (Cell Signaling Technology; #12000 and #12001; Danvers, MA, USA) according to the manufacturer’s instructions. Briefly, the replicates of 100 µg protein lysate (in 100 µL each) were incubated with Smad2/3 mouse antibody coated on the microwells overnight at 4 °C. Following extensive washing, either an SMAD2/3 detection antibody or a phospho-SMAD2 (Ser465/467)/Smad3 (Ser423/425) detection antibody was added to the plate at 37 °C for 1 h to detect the captured protein. The detection antibody was recognized by an anti-rabbit IgG, HRP-linked antibody for 30 min at 37 °C and developed by horseradish peroxidase (HRP) substrate TMB (3,3′,5,5′-tetramethylbenzidine) after 30 min incubation at 25 °C. The absorbance was read at 450 nm within 30 min after the reaction.

#### 2.1.11. MAPK and p-MAPK Assay

The expression levels of the ERK1/2, JNK and P38 and their phosphorylated forms (ERK1/2 (T202/Y204), JNK (T183/Y185), P38 (T180/Y182) were measured using RayBio^®^ Phospho-ERK/JNK/P38α ELISA Kit (#PEL-MAPK-SK, RayBio; GA, USA) as recommended by the manufacturer. Briefly, the replicates of 100 µg protein lysate (in 100 µL each) were incubated with ERK1/”, JNK and P38 antibodies coated on the microwells overnight at 4 °C. The detection antibody was added to each well and incubated with HRP-conjugated anti-rabbit IgG against anti phosphorylated ERK1/2(T202/Y204), JNK (T183/Y185) and P38(T180/Y182) antibodies, or HRP-conjugated Streptavidin against biotinylated anti total ERK1/2, JNK and P38 antibodies, were added to the corresponding wells. The reaction was incubated for 1h at room temperature with shaking. A TMB substrate was added at the end for 30 min at room temperature. The absorbance was read at 450 nm, immediately after adding the STOP solution.

#### 2.1.12. Serum Inflammatory Molecules Analysis

The expression levels of different mediators, including TNF-α, TGF-β, IL-1α and β, IL-6, and interferon (IFN) γ, were measured using ELISA strip colorimetric kits #EA-1401, and #EA-1131 (Signosis, Inc., Santa Clara, SA, USA) according to the manufacturer’s instructions. Briefly, the replicates of 100 µg serum protein (in 100 µL each) were incubated in a microplate coated with the corresponding antibody for 2 h at room temperature with gentle shaking. Following extensive washing steps, the reaction was incubated with 100 μL of diluted biotin-labelled antibody mixture for 1h at room temperature. In each well, 100 μL of the streptavidin-HRP conjugate was added, and they were incubated for 45 min at room temperature. The substrate was added at the end for 30 min at room temperature. The absorbance was read at 450 nm immediately after adding the STOP solution.

#### 2.1.13. Endothelial Nitric Oxide Synthase (eNOS) Activity

The activity of eNOS was compared in mice hearts using a fluorometric assay (ab211084; Abcam; Boston, MA, USA) as recommended by the manufacture. Briefly, the protein was extracted by adding NOS Assay Buffer (containing protease inhibitor cocktail) to the tissue and cold homogenization. The replicates of 200 µg protein (in 40 µL) were incubated with a reaction mix buffer containing NOS cofactor 1 and 2, NOS substrate and nitrate reductase at 37 °C for 1 h. After adding the NOS assay buffer and enhancer, the reaction was incubated at room temperature for 10 min. The fluorescent probe and NaOH were added to all samples which were incubated for a further 10 min before measurement. NO generated by NOS undergoes a series of reactions and reacts with the fluorescent probe to generate a stable signal at Ex/Em = 360/450 nm, which is directly proportional to NOS activity.

#### 2.1.14. Nitric Oxide (NO) Assay

The levels of NO in serum were measured using (#262-200; Biovison, CA, USA) according to the manufacturer’s recommendations. Briefly, the replicates of 100 µg serum protein (in 85 µL each) were incubated in a microplate with 115 μL of the assay buffer and 5 μL of the Nitrate Reductase and 5 μL of the enzyme cofactor, for 1 h at room temperature to convert nitrate to nitrite. The reaction was further mixed with 5 μL of the enhancer and 50 μL of Griess Reagent 1 and 2 and incubated for 10 min at room temperature to convert nitrite to the azo compound. The absorbance was read at 540 nm. The amount of the azo-chromophore accurately reflects the amount of nitric oxide in samples.

#### 2.1.15. Statistical Analysis

GraphPad Prism 5.0 was used for all statistical analyses; a two-tailed unpaired Student t-test was used to compare two groups. Statistical significance was accepted when *p* < 0.05. Data are presented in figures as means of biological replicates ±SEM.

#### 2.1.16. Data Availability

The raw MS data can be accessed from the STOREDB database Available online: doi:10.20348/STOREDB/1171 ((accessed on 14 October 2021).

## 3. Results

### 3.1. The Proteins Associated with Cardiac Metabolism Were Changed after Treatment with Fenofibrate

**Figure 1:**
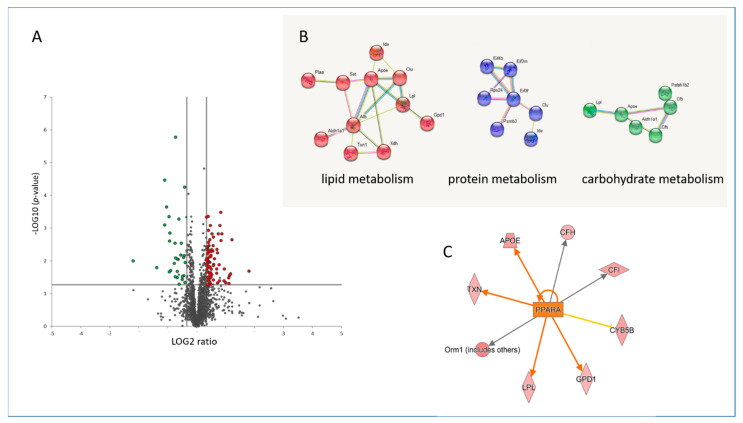
Changes in the cardiac proteome after treatment with fenofibrate. Volcano plot represents the distribution of all quantified proteins in cardiac tissue after treatment with fenofibrate. Deregulated proteins (+1.3-fold; *p* < 0.05) are highlighted in green (downregulated) and red (upregulated) (**A**). Protein–protein interactions were analyzed by the STRING software tool. Available online: (http://string-db.org) (accessed on 14 October 2021). indicating the most affected protein clusters (**B**). Prediction of activation of PPARα (orange colour) based on deregulated proteins. The upregulated proteins are marked in red (**C**). The analyses were generated using Ingenuity^®^ Pathway Analysis. Available online: (https://www.qiagenbio-informatics.com/products/ingenuity-pathway-analysis) (accessed on 14 October 2021). The full protein names are provided in Tables S1 and S2.

The cardiac proteome of mice treated with fenofibrate was compared to that of placebo-treated mice, using label-free proteomics. Among the 1561 quantified proteins with at least 2 unique peptides (Table S1), the expression of 108 proteins was changed significantly (±1.3-fold; *p* < 0.05) after treatment with fenofibrate. Out of those, 78 proteins were down- and 30 were up-regulated (Figure 1A and Table S2). More than half (52%) of the significantly deregulated proteins were involved in the metabolic process, including the protein, lipid and carbohydrate metabolism (Figure 1B and Table S3). PPARα, as a key transcription factor of lipid metabolism, was predicted to be activated after fenofibrate treatment (Figure 1C and Table S3), showing that this ligand is able to activate cardiac PPARα in vivo if orally administered to mice.

### 3.2. Fenofibrate Reduces the Effect of Local High-Dose Irradiation on the Cardiac Proteome

**Figure 2:**
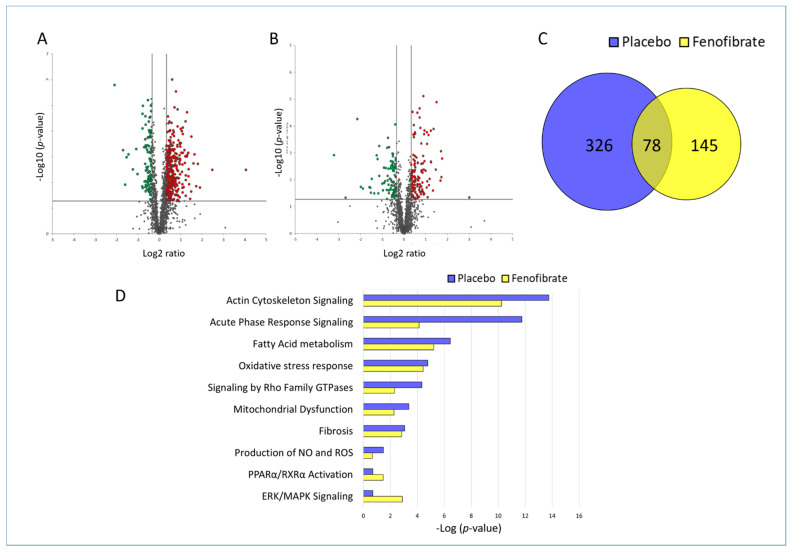
Changes in the proteome of irradiated heart with and without fenofibrate treatment. Volcano plots represent the distribution of all quantified proteins in irradiated cardiac tissue without (**A**) or with (**B**) fenofibrate. Deregulated proteins (±1.3-fold; *p* < 0.05) are highlighted in green (downregulated) and red (upregulated). The full protein names are provided in Table S1. A Venn diagram illustrating the shared deregulated proteins between the two experimental groups (**C**). The most significant canonical pathways altered in both proteome profiles (**D**). The analyses were generated by Ingenuity^®^ Pathway Analysis. Available online: https://www.qiagenbio-informatics.com/products/ingenuity-pathway-analysis) (accessed on 14 October 2021). Bars indicate canonical pathways with the enrichment significance shown on the horizontal axis (-log *p*-value). Longer bars indicate a higher significance of the pathway.

The effect of irradiation on the cardiac proteome was compared in fenofibrate- and placebo-treated mice. The list of all identified and quantified proteins is available in Supplementary Table S1. The analysis showed that irradiation altered the expression level of 404 (311 up- and 93 down-regulated) and 223 (131 up- and 92 down-regulated) proteins (±1.3-fold; *p* < 0.05) in placebo- and fenofibrate-treated mice, respectively (Figure 2A,B and Tables S4 and S5). Importantly, the reduction in the number of dysregulated proteins in fenofibrate-treated mice compared to those treated by placebo suggested that PPARα activation potentially improves the cardiac proteome response to irradiation. Among the significantly deregulated proteins, 78 proteins were shared by both treatments in irradiated hearts (Figure 2C and Tables S4 and S5). These shared proteins were mainly involved in stress response and cytoskeleton organization, suggesting a general response to radiation exposure (analyzed by the STRING software tool. Available online: (http://string-db.org) (accessed on 14 October 2021). 

The protein–protein interactions and affected biological pathways were predicted using Ingenuity Pathway Analysis (IPA) software. Available online: (https://www.qiagenbio-informatics.com/products/ingenuity-pathway-analysis) (accessed on 14 October 2021). The comparison of the most affected pathways in the heart of placebo- and fenofibrate-treated mice indicated that treatment with fenofibrate reduced the significance of enriched signalling pathways such as actin cytoskeleton, acute phase response, fatty acid metabolism, stress response, mitochondrial dysfunction, production of NO and ROS, and fibrosis (Figure 2D and Table S6). In contrast, more proteins were enriched in the glycolysis, PPARα /RXR and ERK signalling pathways after fenofibrate treatment in irradiated hearts, suggesting the potential induction of these pathways (Figure 2D and Table S6).

### 3.3. Fenofibrate Restored the Effect of Irradiation on PPARα and Its Target Genes in Lipid Metabolism in Sham-Irradiated Heart

**Figure 3:**
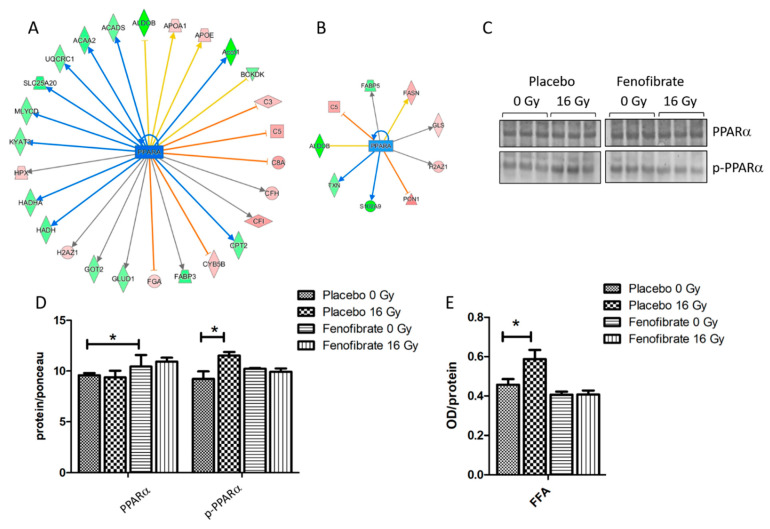
Analysis of the cardiac PPARα and serum FFA. Graphical representations of the deregulated protein networks with their upstream transcriptional regulators PPARα in the irradiated heart after treatment with placebo (**A**) and fenofibrate (**B**). The upregulated proteins are marked in red and the downregulated are marked in green. The nodes in blue (inhibition) and orange (activation) represent transcription factors. The analyses were generated using Ingenuity^®^ Pathway Analysis. Available online: https://www.qiagenbio-informatics.com/products/ingenuity-pathway-analysis) (accessed on 14 October 2021). The full protein names are given in Table S1. Immunoblot analysis of PPARα was performed in mouse hearts (**C**). The amount of total protein was measured by Ponceau S staining. The columns represent the average ratios of relative protein expression in control and irradiated samples after background correction (±SEM) (*t*-test; * *p* < 0.05; n = 3) (**D**). The amount of the FFA was measured in 100 µg of serum using ELISA (+SEM) (*t*-test; * *p* < 0.05; n = 5) (**E**).

Proteomics data indicate that several enzymes of the lipid metabolism associated with PPARα were significantly downregulated in the heart following irradiation (Figure 3A and Table S6). The number of PPARα-associated proteins were found to be reduced after fenofibrate treatment (Figure 3B). Since the activity of PPARα is regulated by phosphorylation [11,34], the expression levels of PPARα full-length protein and its phosphorylated form were measured. The expression level of PPARα remained unchanged after irradiation, as previously observed (references mouse and male) but significantly increased after fenofibrate treatment. The expression level of deactivated (phosphorylated) PPARα was increased following irradiation in placebo-treated mice, while this effect was prevented by fenofibrate (Figure 3C,D).

Furthermore, the serum of mice was tested for levels of free fatty acids (FFA). In good agreement with previous data [11,28], the level of free fatty acids was increased in the serum after local heart exposure (16 Gy) (Figure 3E) suggesting a radiation-induced impairment of the fatty acid metabolism. In the fenofibrate-fed mice, the amount of serum FFA returned to normal levels (Figure 3E).

### 3.4. PPARα Activation Ameliorates the Alterations in the Mitochondrial Proteins and OXPHOS Activity in Irradiated Hearts

**Figure 4:**
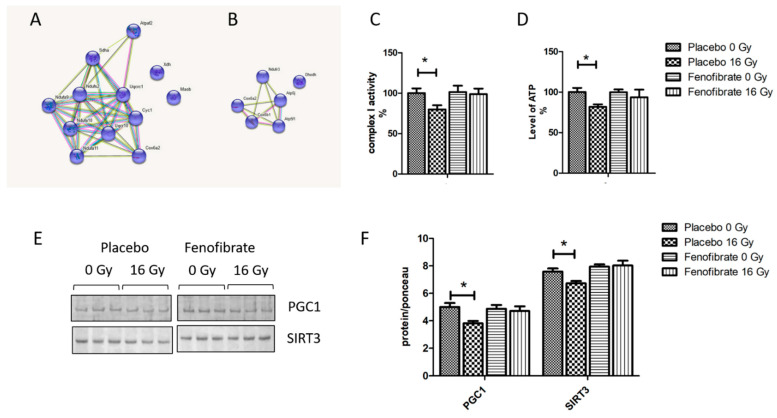
The effect of fenofibrate on mitochondria of irradiated heart. The cluster of deregulated mitochondrial proteins in the irradiated heart without (**A**) and with (**B**) fenofibrate treatment. Analysed by STRING software. Available online: (http://string-db.org) (accessed on 14 October 2021). The activity of complex I (**C**) and the level of cardiac ATP (**D**) were measured and expressed as a percentage compared to sham-irradiated, placebo-treated mice. The error bars represent standard error of the mean (±SEM) (*t*-test; * *p* < 0.05; n = 5). Immunoblot analysis of PGC1 and SIRT3 (**E**). The amount of the total protein was measured by Ponceau S staining for an accurate comparison between the groups. The columns represent the average ratios of relative protein expression in control and irradiated samples (**F**). The error bars represent standard error of the mean (±SEM) (*t*-test; * *p* < 0.05; n = 3).

Comparison of deregulated mitochondrial proteins in the proteome of mice treated with placebo or fenofibrate demonstrate that their number was reduced after fenofibrate treatment in irradiated mice (Figure 4A,B). To investigate the effect of the fenofibrate on radiation-induced mitochondrial impairment, we examined mitochondrial complex I activity and the ATP level. An analysis showed that the activity of respiratory complex I and the amount of ATP decreased by almost 20% following irradiation. However, fenofibrate treatment restored the changes to the normal level (Figure 4C,D). Fenofibrate also restored the radiation-induced downregulation of PGC1 and SIRT3, which act as key regulators of mitochondrial biogenesis and metabolism (Figure 4E,F).

### 3.5. Radiation-Induced Oxidative Stress Was Improved by PPARα Activation

**Figure 5:**
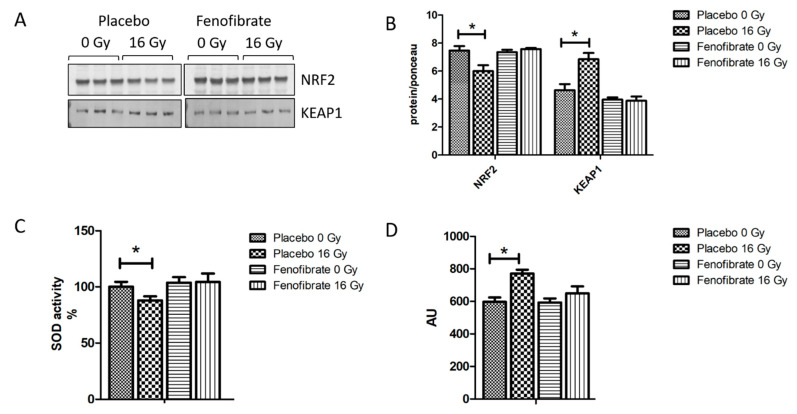
Analysis of the oxidative stress response. Immunoblot analysis of NRF2 and KEAP1 is shown (**A**). The amount of the total protein was measured by Ponceau S staining. The columns represent the average ratios of relative protein expression in control and irradiated samples after background correction (*t*-test; * *p* < 0.05; n = 3) (**B**). The activity of SOD2 was compared in (**C**). The amount of lipid peroxidation as a marker of oxidative stress was measured in mice hearts (**D**). The error bars represent standard error of the mean (±SEM) (*t*-test; * *p* < 0.05; n = 3).

Impairment of the mitochondrial respiratory complexes contributes to the formation of reactive oxygen species and thereby increases oxidative stress [32]. The proteomics data suggested that the activation of PPARα reduced radiation-induced oxidative stress. To validate this finding, the expression levels of nuclear factor erythroid 2-related factor 2 (NRF2), the main regulator of the oxidative stress response, its repressor Kelch ECH associating protein 1 (KEAP1), the activity of SOD (oxidative response enzyme) and the level of malondialdehyde modification (oxidative stress marker) were measured using immunoblotting and ELISA. A marked decrease in the expression level of NRF2 (Figure 5A,B), increased KEAP1, reduced SOD activity (Figure 5C), and increased levels of lipid peroxidation (Figure 5D) were observed in irradiated hearts. However, these levels were returned to normal when mice were treated with fenofibrate (Figure 5A–D).

### 3.6. PPARα Activation Decreased the Systematic Inflammatory Response Following Irradiation

**Figure 6:**
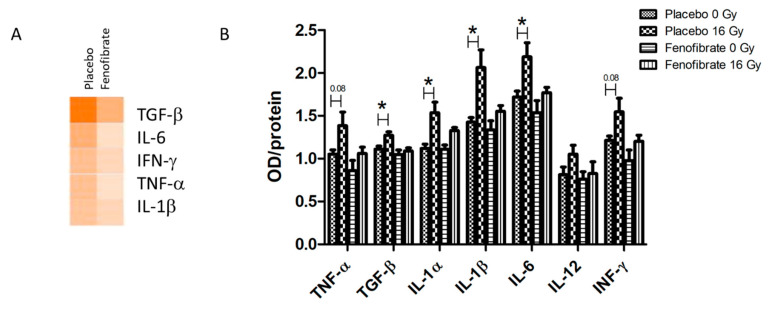
The analysis of serum cytokines. Graphical representations of the potential upstream transcriptional regulators predicted to be affected in the irradiated heart after treatment with placebo and fenofibrate (**A**). The predictions were generated based on the deregulated proteins using Ingenuity^®^ Pathway Analysis. Available online: (https://www.qiagenbio-informatics.com/products/ingenuity-pathway-analysis) (accessed on 14 October 2021). The scores of activities are displayed using an orange colour gradient where a darker colour corresponds to a high score (high statistical significance) (**A**). The level of cytokines was measured in 100 µg of serum using ELISA. The error bars represent standard error of the mean (±SEM) (*t*-test; * *p* < 0.05; n = 5) (**B**).

The inflammatory mediators were predicted to become upregulated in the heart proteome following local irradiation (Figure 6A and Table S6). The levels of seven different cytokines and inflammatory mediators were measured in the serum of mice using ELISA. In good agreement with our previous data [28], the levels of TGF-β, IL-1 α and β, and IL-6, were significantly increased following irradiation (16 Gy) in comparison to the controls (Figure 6B). Importantly, when using fenofibrate treatment the levels of pro-inflammatory cytokines were restored to the levels observed in controls (Figure 6B).

### 3.7. Fenofibrate Attenuated the Up-Regulation of Proteins Involved in Tissue Remodelling in Irradiated Heart

**Figure 7:**
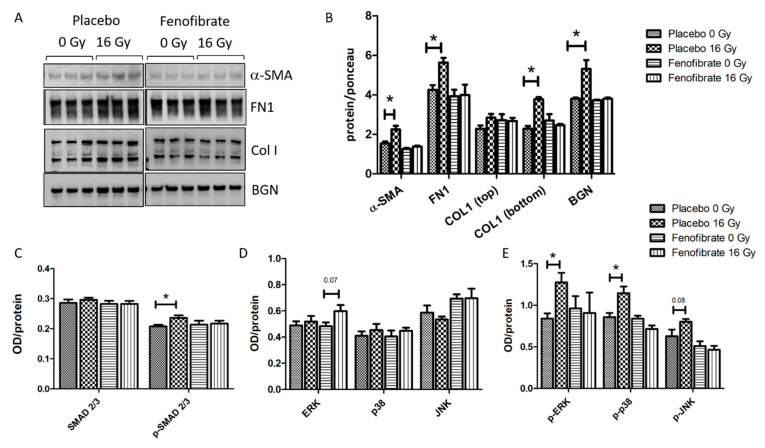
Analysis of the ECM markers and TGF-β signalling pathways. Immunoblot analysis of ECM markers is shown (**A**). The amount of the total protein was measured by Ponceau S staining. The columns represent the average ratios of relative protein expression in control and irradiated samples after background correction (*t*-test; * *p* < 0.05; n = 3) (**B**). The expression levels of SMAD 2/3 and p-SMAD 2/3 were compared in mice hearts using ELISA (**C**). The expression levels of ERK, p38, JNK and their phosphorylated forms were compared in mice hearts using ELISA (**D**,**E**). The error bars represent standard error of the mean (±SEM) (*t*-test; * *p* < 0.05; n = 3).

The effect of fenofibrate in counteracting the upregulation of TGF-β in the serum of irradiated mice prompted us to examine whether the activation of PPARα attenuates radiation-induced tissue remodeling and fibrosis. Therefore, the expression levels of alpha-smooth muscle actin (α-SMA), a marker of fibroblast differentiation, as well as fibronectin (FN1), collagen I (COLI) and biglycan (BGN)—markers of tissue fibrosis—were measured using immunoblotting. The analysis demonstrated that the levels of α-SMA, FN1, COLI and BGN were increased in irradiated hearts, whereas treatment with fenofibrate could reverse these changes and return them to the control levels (Figure 7A,B).

The activation of canonical (SMAD) and non-canonical (MAPK) TGF-β signalling are understood to be the key pathways involved in the initiation of fibrosis [35,36]. In agreement with previous data [12,13], radiation exposure was found to be associated with an increase in the ratio of activated (phosphorylated) SMAD 2/3 to total SMAD 2/3 levels. The treatment with fenofibrate prevented the radiation-induced increase in p-SMAD levels in irradiated hearts (Figure 7C). Similarly, and as shown before [12,13], the expression levels of components of the TGF-β non-canonical pathway, such as p-ERK, p-p38 and p-JNK, were increased following irradiation (Figure 7D,E). Again, fenofibrate treatment returned the level of active (phosphorylated) MAPK proteins to those of the control levels (Figure 7E).

### 3.8. PPARα Activation Restored the Effect of Irradiation on eNOS and NO Production

**Figure 8:**
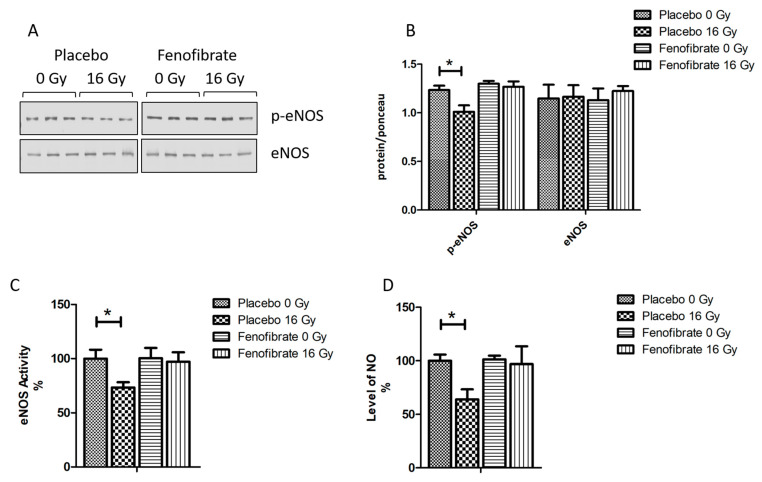
Analysis of the cardiac eNOS and serum NO. Immunoblot analysis of eNOS was performed in mouse hearts (**A**). The amount of the total protein was measured by Ponceau S staining. The columns represent the average ratios of relative protein expression in control and irradiated samples after background correction (±SEM) (*t*-test; * *p* < 0.05; n = 3) (**B**). The activity of eNOS was compared in mice hearts (**C**). The levels of NO were compared in mice serum (**D**). The error bars represent standard error of the mean (±SEM) (*t*-test; * *p* < 0.05; n = 3).

The impairment of nitric oxide (NO) signalling has been repeatedly reported for in vitro and in vivo models after exposure to moderate to high doses of radiation [37,38,39]. Proteome profiling indicated an effect of radiation on NO production (Figure 2D and Table S6). To investigate the effect of fenofibrate on the NO signalling, we measured the expression level and activity of endothelial nitric oxide synthase (eNOS), a marker of vascular functionality in the heart, and the level of NO in the serum of all mice. The expression level of the phosphorylated (active) form of eNOS and its activity was reduced in the cardiac tissue following irradiation (Figure 8A–C). The level of NO in the serum was also significantly reduced in irradiated mice (Figure 8D). However, these changes were reversed when the mice were treated with fenofibrate (Figure 8A–D).

## 4. Discussion

The present study was designed to investigate the potential protective effects of fenofibrate, a ligand of PPARα, on radiation-induced heart damage. We previously demonstrated that radiation-induced cardiac injury is associated with the impairment of the cardiac metabolism [8,9,10] and a persistent reduction in the activity of cardiac PPARα and its related signalling pathways [11,12,13].

To the best of our knowledge, this is the first study to have investigated the effects of fenofibrate treatment on the cardiac proteome after local high-dose irradiation. The study showed that radiation-induced alterations such as a reduced mitochondrial complex activity increased oxidative stress, the increased inflammatory response and the induction of pathways involved in cardiac fibrosis were all reversed in fenofibrate-treated mice.

Fenofibrate is a well-known hypolipidemic drug that has been used to treat hypertriglyceridemia, hypercholesterolemia and mixed dyslipidemia in patients [40,41]. The beneficial effects of PPAR ligands have been demonstrated on various cardiovascular risk factors [42,43,44,45]. Agonist activation of PPARα has been shown to protect against acute myocardial ischemia [46], myocardial remodeling and hypertension [47], hypertrophy [48], diabetic cardiomyopathy [49], atherogenesis [50] and vascular injury [44]. The outcome profiles of clinical trials using different PPARα agonists were not consistently similar. Further studies are still required to achieve an optimal and harmonized outcome profile for the clinical administration of PPARα agonists.

The activity of the PPARα transcription factor is tightly regulated at post-translational levels [51]. Phosphorylation affects the stability of the PPAR transcription machinery due to the reduced binding capacity of PPARα in the transcriptional coactivators, corepressors or ligands [15,34]. Here, we showed that fenofibrate reduced phosphorylation and thereby counteracted the radiation-induced inactivation of PPARα. This was observed for several PPARα-associated parameters, for example in the restoration of the serum FFA level, which was increased after irradiation. Elevated circulating FFA levels are a marker of impaired myocardial fatty-acid oxidation (FAO) or fatty-acid uptake, both of which are regulated by PPARα [52,53]. PPARα-null mice exhibit significantly lower expression of proteins involved in FA metabolism and transport. The improvement in PPAR-related proteins presented here strongly suggests a preventive effect of fenofibrate on radiation-induced cardiac metabolic impairment.

We previously reported that irradiation induces persistent functional and proteome alterations in cardiac mitochondria that correlate with the reduced activity of proteins of the respiratory chain [10,54,55]. Mitochondria, which comprise nearly 40% of the total volume of normal cardiomyocytes, are key components in the generation of ATP for contractile function [56]. The impairment of the mitochondrial function is tightly linked to cardiac disease [57]. In the present study, we observed that fenofibrate treatment reduced the number of deregulated mitochondrial proteins and improved the activities of complex I and the level of cardiac ATP in irradiated mice. The radiation-induced downregulation of PGC1 and SIRT3, which are two of the key regulators of mitochondrial biogenesis and homeostasis, was also attenuated in the fenofibrate-treated mice. The role of the PPARα transcription complex and the PPAR coactivator PGC-1 in mitochondrial OXPHOS has been well described [58,59]. The regulatory role of the SIRT/PGC-1/PPAR-alpha network in the maintenance of cardiac metabolism has also been described [60,61,62]. PPARα-null mice show a downregulation of genes related to OXPHOS [63]. In agreement with our data, PPARα activation by fenofibrate has previously been shown to improve the mitochondrial oxidative capacity, induce fatty-acid oxidation, and reduce mitochondrial apoptosis and membrane depolarization [48,64].

Radiation-induced mitochondrial dysfunction results in the enhancement of reactive oxygen species (ROS) production [10,55] and the level of oxidative stress-induced protein modifications [11,37] resulting in the deactivation or degradation of proteins [65,66]. We found that radiation-induced oxidative stress was decreased in fenofibrate-treated mice, by improving the level of NRF2, a key transcription factor of oxidative stress and the activity of SOD2. The long-term downregulation of several oxidative stress proteins and inactivation of NRF2 was observed in locally irradiated mice hearts [13] and in the heart proteome of Mayak workers following chronic exposure in the highest dose group (<500 mGy) [24]. Interestingly, PPARα-null mice exhibited marked oxidative and nitrosative modifications of contractile proteins [67]. Similarly, fenofibrate prevented the increased the levels of oxidative stress modifications in the irradiated hearts. We believe that this may be a direct effect on the activation of PPARα, which is known to modulate cellular-redox homeostasis and to activate antioxidant defence [68,69], and fenofibrate treatment has been shown to increase the level of NRF2 and reduce MDA for in vitro and in vivo models [70,71].

The impairment of the PPARα signalling pathway, as observed in irradiated hearts, might be involved in the alteration of the cardiac oxidation/reduction equilibrium and may result in chronic inflammation [67]. Our data showed that the radiation-induced increase in the inflammatory response [28,37,72,73] was prevented in fenofibrate-treated mice. The activation of PPARα has been shown to reduce the production of several inflammatory genes or proteins including TNF-α, IL-1β and IL-6, suggesting a reduction in the systemic inflammatory response [17,18,74]. Radiation-induced cardiac damage is accompanied by tissue remodeling and fibrosis [13,75,76]. We have previously shown that the interplay between PPARα and TGF-β contributes to structural remodeling and fibrosis in the irradiated heart [12,13]. The regulatory interplay between PPARα and TGF-β has previously been described [77,78]. Agonist-activated PPARα has been shown to inhibit the TGF-β non-canonical pathway by suppressing MAPK/ JNK [78,79], or by inhibiting the phosphorylation of TGF-β activated kinase 1 (TAK1), resulting in reduced fibrosis [80]. In good agreement with these findings, data presented here indicated that the activation of PPARα inhibits the induction of the TGF-β signalling pathway and the level of expression of ECM markers in irradiated mice. This is in agreement with previous data showing that PPARα activators counteract cardiac remodeling and fibrosis [79,81,82].

Endothelial dysfunction is involved in radiation-induced cardiac vascular damage [83,84,85]. We previously demonstrated that local high-dose irradiation of the heart (16 Gy) decreases NO levels in the serum of mice due to the reduced phosphorylation of eNOS in cardiac endothelial cells [37], which are pathological events known to be hallmarks of endothelial dysfunction and of impaired endothelium-dependent vasodilation [86,87,88]. The disruption of NO pathway is associated with metabolic disordering, mitochondrial dysfunction and elevated free fatty-acid concentrations [89,90]. In good agreement with previous data [37,38,39], we observed that the radiation-induced reduction in eNOS activity and NO production were attenuated in fenofibrate-treated mice. The activation of PPARα has been reported to improve the endothelial function, eNOS expression and NO production [91,92,93,94].

Taken together, these findings highlight the central role of PPARα in orchestrating the cardiac response to high-dose radiation. The activation of PPARα seems to reverse the adverse outcomes of irradiation on the heart [95] by influencing the expression of key proteins in mitochondrial biogenesis and homeostasis, restoring metabolic pathways, reducing fibrosis and tissue remodeling and re-establishing the NO balance in the heart.

## 5. Conclusions

To the best of our knowledge, the present study is the first quantitative analysis of the cardiac proteome of fenofibrate-treated mice after their exposure to local high-dose ionizing radiation. The data presented here indicate that the fenofibrate treatment ameliorates the adverse effects of irradiation in the heart proteome. These findings suggest that the administration of PPARα agonists could be particularly beneficial in radiotherapy affecting the thorax region, where the heart may receive significant radiation doses, leading to adverse cardiovascular events.

## Data Availability

The raw MS data can be accessed from the STOREDB database Available online: DOI:10.20348/STOREDB/1171 (accessed on 14 October 2021).

## Supplementary Materials

The following are available online at https://www.mdpi.com/article/10.3390/biomedicines9121845/s1. Figure S1. Full-length images of Ponceau S staining and antibody detections of replicates for PGC1, p-PPARα and SIRT3. Figure S2. Full-length images of Ponceau S staining and antibody detections of replicates for NRF2 and α-SMA. Figure S3. Full-length images of Ponceau S staining and antibody detections of replicates for FN1, Col I and BGN. Figure S4. Full-length images of Ponceau S staining and antibody detections of replicates for p-eNOS and KEAP1. Figure S5. Full-length images of Ponceau S staining and antibody detections of replicates for PPARα and eNOS. Table S1. All identified and quantified proteins. Table S2. Significantly dergulated proteins in sham-irradiated heart after treatment with Fenofibrtae. Table S3. Main affected molecular functions and upstream regulators in sham-irradiated heart after treatment with Fenofibrtae. Table S4. Significantly dergulated proteins in irradiated heart treated with placebo. Table S5. Significantly dergulated proteins in irradiated heart treated with Fenofibrate. Table S6. Comparison of the main affected pathways and upstream regulators in irradiated heart with placebo and fenofibrate.
