# Supplementary material for: Activation of PPARα by Fenofibrate Attenuates the Effect of Local Heart High Dose Irradiation on the Mouse Cardiac Proteome
Source: Biomedicines. 2021 Dec 6;9(12):1845. doi: 10.3390/biomedicines9121845 (PMC8698387; doi:10.3390/biomedicines9121845)
Supplement: Supplementary file 1 [file biomedicines-09-01845-s001.zip › biomedicines-1441126-supplementary.pdf]

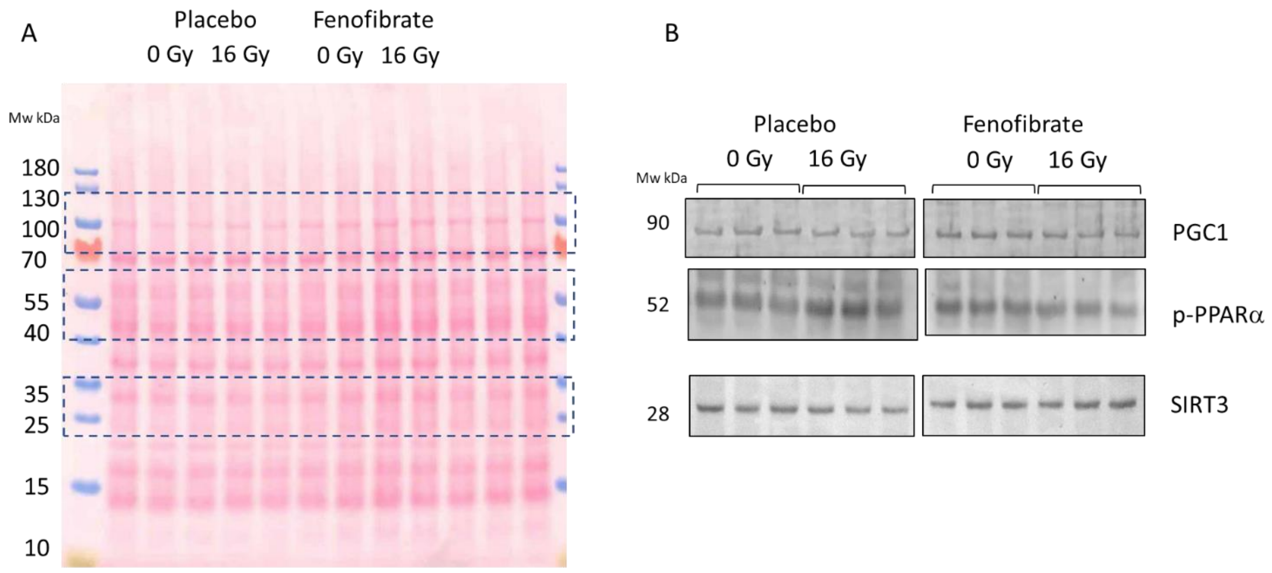

Figure S1. Full-length images of Ponceau S staining and antibody detections of replicates for PGC1, p-PPARα and SIRT3.

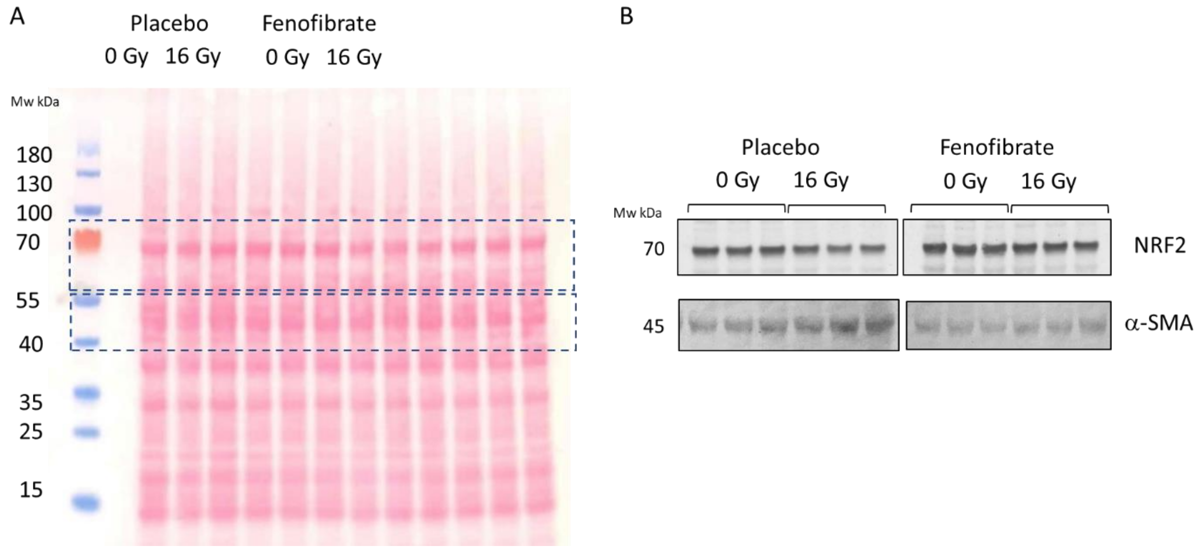

Figure S2. Full-length images of Ponceau S staining and antibody detections of replicates for NRF2 and α-SMA.

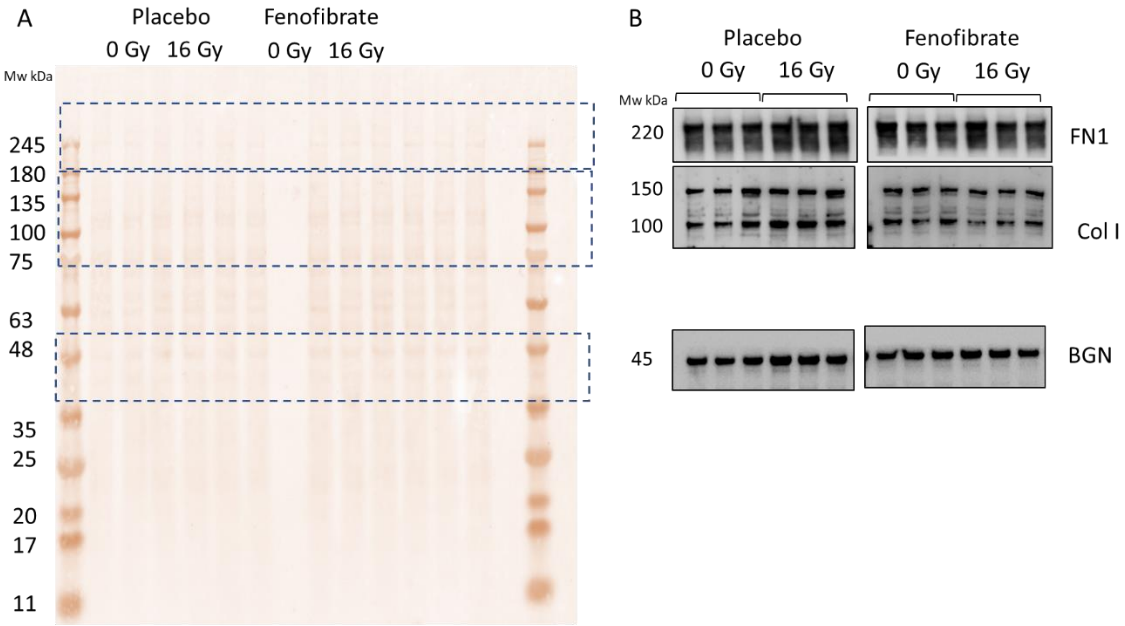

Figure S3. Full-length images of Ponceau S staining and antibody detections of replicates for FN1, Col I and BGN.

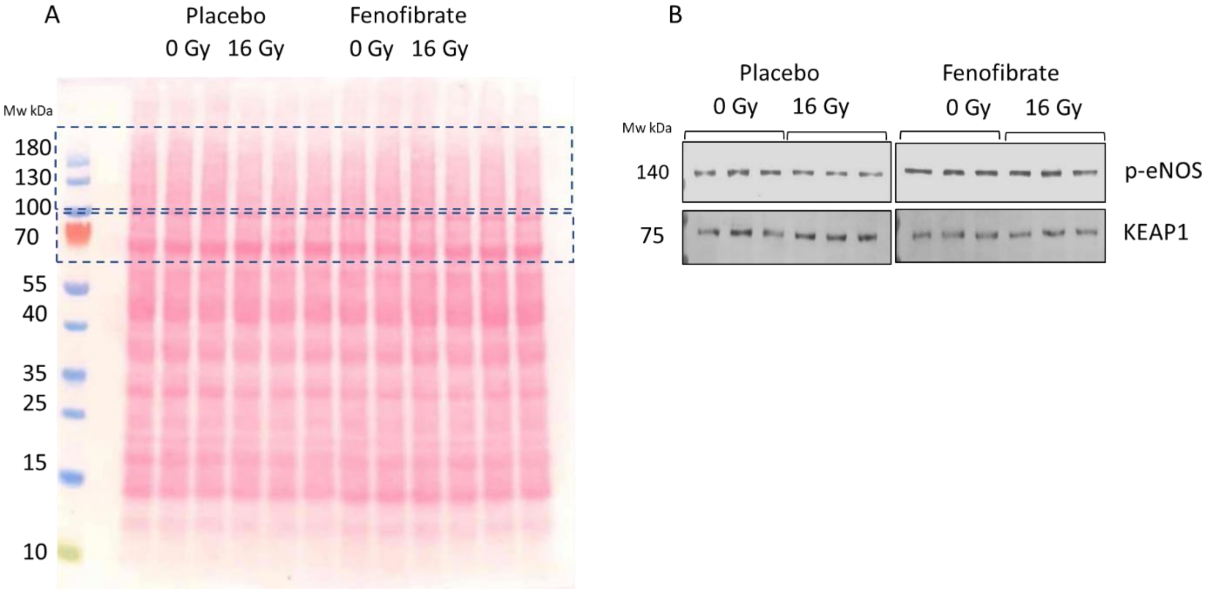

Figure S4. Full-length images of Ponceau S staining and antibody detections of replicates for p-eNOS and KEAP1.

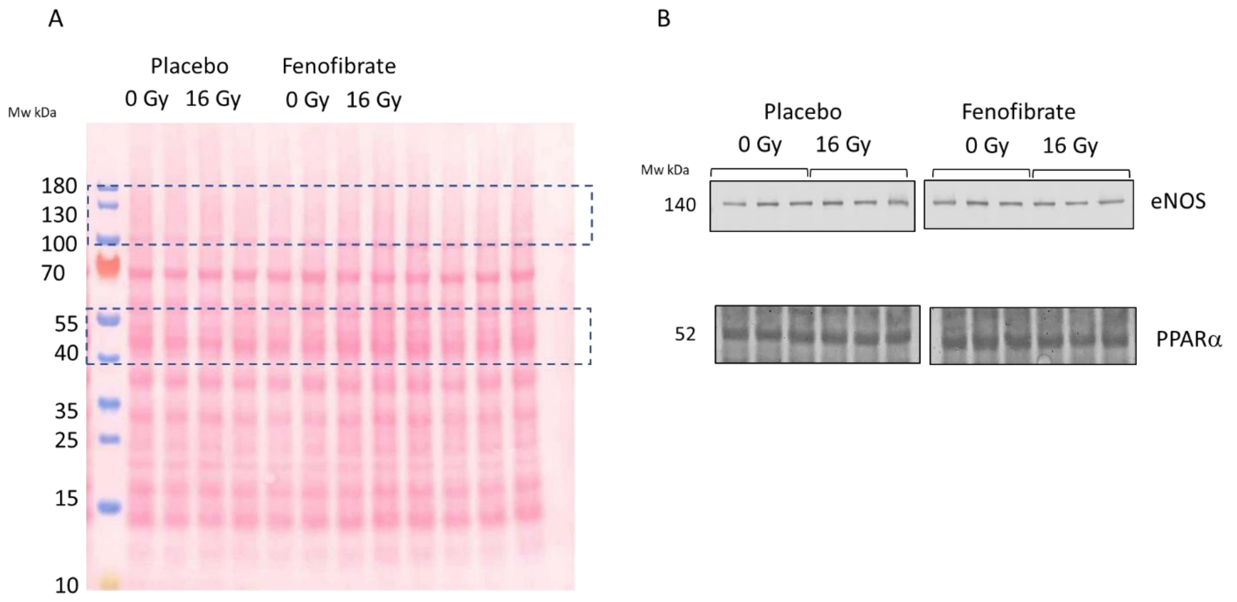

Figure S5. Full-length images of Ponceau S staining and antibody detections of replicates for PPAR α and eNOS
